# Association of quantitative measures of effusion-synovitis and hoffa-synovitis with radiographic and pain progression: Data from the FNIH OA biomarkers consortium

**DOI:** 10.1016/j.ocarto.2021.100138

**Published:** 2021-01-11

**Authors:** Stacy E. Smith, Shayan Hosseinzadeh, Troy Maetani, Pandey Shilpa, Jamie E. Collins, C. Kent Kwoh, Jeffrey Duryea

**Affiliations:** a Division of Musculoskeletal Imaging and Intervention, Department of Radiology, Brigham and Women’s Hospital, Harvard Medical School, Boston, MA, USA; b Department of Radiology, Brigham and Women’s Hospital, Harvard Medical School, Boston, MA, USA; c Neil and Elise Wallace STRATUS Center for Medical Simulation, Brigham and Women’s Hospital, Harvard Medical School, Boston, MA, USA; d Children’s Hospital, Harvard Medical School, Boston, MA, USA; e Department of Orthopedics, Brigham and Women’s Hospital, Harvard Medical School, Boston, MA, USA; f University of Arizona Arthritis Center, The University of Arizona College of Medicine, Tucson, AZ, USA

**Keywords:** Effusion, Synovitis, Segmentation, MRI, Osteoarthritis, Knee

## Abstract

**Objective:**

To validate a semi-automated software method of quantifying knee osteoarthritis (KOA) related effusion-synovitis (ES) and Hoffa-synovitis (HS) on MRI.

**Materials and methods:**

301 subjects were randomly selected from the FNIH sub cohort, a nested case control study within the Osteoarthritis Initiative (OAI), and distributed into 4 groups based on pain and radiographic progression. Measurements of ES and HS volume were made by 2 readers. Criterion validation was assessed through comparison with the MRI Osteoarthritis Knee Score (MOAKS) and the Spearman correlation coefficient r value. Reader reliability was measured on a subset of 30 subjects and intra-class correlation coefficients (ICCs). Clinical validity was assessed based on case control status using logistic regression and the area under the curve (AUC).

**Results:**

ES volume was highly correlated with MOAKS Scores (r ​= ​0.74), as was the HS measure but to a lesser extent (r ​= ​0.55). For ES, the intra-reader and intra-reader precision ICCs were 0.83 and 0.95 respectively and 0.98 and 0.96 for HS. For clinical validity, we found similar AUC values when comparing the software method to MOAKS. The average reader time was less than 15 ​min per knee for both ES and HS.

**Conclusion:**

We have demonstrated the validity of an efficient, accurate, and rapid ES and HS measurement method for KOA using MRI. To our knowledge, this is the first such software to measure both ES and HS. This method will offer an objective and efficient tool for clinical trials and other epidemiologic studies of KOA.

## 1 Introduction

Osteoarthritis (OA) is the most common type of arthritis worldwide, resulting in pain, joint deformity and disability [[1], [2], [3]]. Therapies targeting specific tissues of disease pathogenesis in OA, including bone, menisci and synovium are of great interest. Inflammation is demonstrated in the majority of individuals with knee OA [4,5] This manifests as synovial membrane thickening/edema within Hoffa’s fat pad inferior to the patella and/or fluid signal intensity and synovial membrane thickening, effusion-synovitis (ES), within the suprapatellar bursa. Both are characterized by high signal intensity edema/fluid on non-contrast fluid sensitive magnetic resonance imaging (MRI) sequences [6,7]. Synovitis is also known to be associated with pain, disease, severity and progression of knee OA [8] and is a known precursor to radiographic OA (ROA) [7]. Both ES (suprapatellar region) and Hoffa synovitis (HS) (infrapatellar/intercondylar region) therefore are felt to represent surrogate markers of inflammation of the synovium in patients with OA. Haradan et al. recently discovered a subset of 6 synovial fluid biomarkers related to synovial inflammation, symptoms and radiographic severity in OA patients, attesting to an inflammatory OA endotype that could prove to be a useful target for future therapies [9].

MRI has proven useful in evaluating tissues involved in OA, particularly for bone marrow lesions (BMLs) [10], meniscus, and hyaline cartilage [11,12]. While qualitative descriptions are commonly used clinically, semi-quantitative scales are a current standard in OA research [13]. Semi-quantitative scoring methods have been developed to assess structural damage in OA include the Whole Organ Magnetic Resonance Imaging Score (WORMS) [14], Boston Leeds Osteoarthritis Knee Score (BLOKS) [15], Knee Osteoarthritis Scoring System (KOSS) [16] and MRI Osteoarthritis Knee Score (MOAKS) [17]. However, these techniques offer ordinal scores and are based on a more subjective assessment of knee MRIs. While large-scale studies of synovium (effusion-synovitis) using MRI and fully quantitative volumetric methods in OA have been performed in 4 prior studies [[18], [19], [20], [21]], these studies evaluated only suprapatellar effusion/synovitis (ES). This is the first study to our knowledge to evaluate both suprapatellar effusion-synovitis (ES) and Hoffa’s-synovitis (HS) using a fully quantitative volumetric method.

While semi-quantitative assessment is useful, a semi-automated quantitative measure of ES volume (software-based methods to segment or outline the area of interest on MR slices) is potentially more objective and granular, and has the potential to be a more efficient alternative to semi-quantitative scoring. Applying quantitative methodology, the software can make measurements of volume and gray scale intensity and report the number of voxels included in the area outlined on an image on a select number of MR slices. In addition, this methodology can potentially be used by properly trained readers with less expertise than a fully trained musculoskeletal radiologist.

The purpose of this paper is to describe and validate a novel and rapid software method for quantification of ES and HS in KOA through comparison with MOAKS scores and to assess clinical validity as well as reader precision.

## 2 Materials and Methods

### 2.1 Study design and cohort

We used subjects from the Osteoarthritis Initiative (OAI), a longitudinal multicenter cohort study of biomarkers and risk factors in 4796 participants between 45 and 79 years of age with or at risk of developing osteoarthritis of the knee. Major exclusionary criteria for the OAI include presence of rheumatoid arthritis, inflammatory arthritis, bilateral end stage knee OA, inability to walk without aids, or a 3 ​T MRI contraindication. The OAI was conducted in compliance with the local Institutional Review Board regulations. Full details of the OAI study are available online (https://nda.nih.gov/oai/). The participants for this study were selected from the baseline visit of the 600 participant Foundation for the National Institutes of Health (FNIH) sub cohort, a nested case-control study within the OAI. Details of this sample have been published [22]. The FNIH study divided the participants into 4 groups based on pain and/or radiographic progression. Following the design of a previous study [23], we randomly selected 301 of the 600 FNIH participants such that the proportion for each group matched the main study as closely as possible. The 301 subjects were distributed as follows:

Group 1: radiographic and pain progressors (n ​= ​97),
Group 2: radiographic-only progressors (n ​= ​52),
Group 3: pain-only progressors (n ​= ​52),
Group 4: no radiographic or pain progressors (n ​= ​100).

Since the number of subjects for Groups 2 and 3 in the full 600 subjects’ study was odd (n ​= ​103), achieving a perfect 50% split was impossible.

The primary case-control analysis [22] defined participants in Group 1 as cases and participants in Groups 2, 3, and 4 as controls. We also investigated two additional different case-control definitions isolating radiographic and pain progression separately.

### 2.2 MRI and software method

**Fig. 1:**
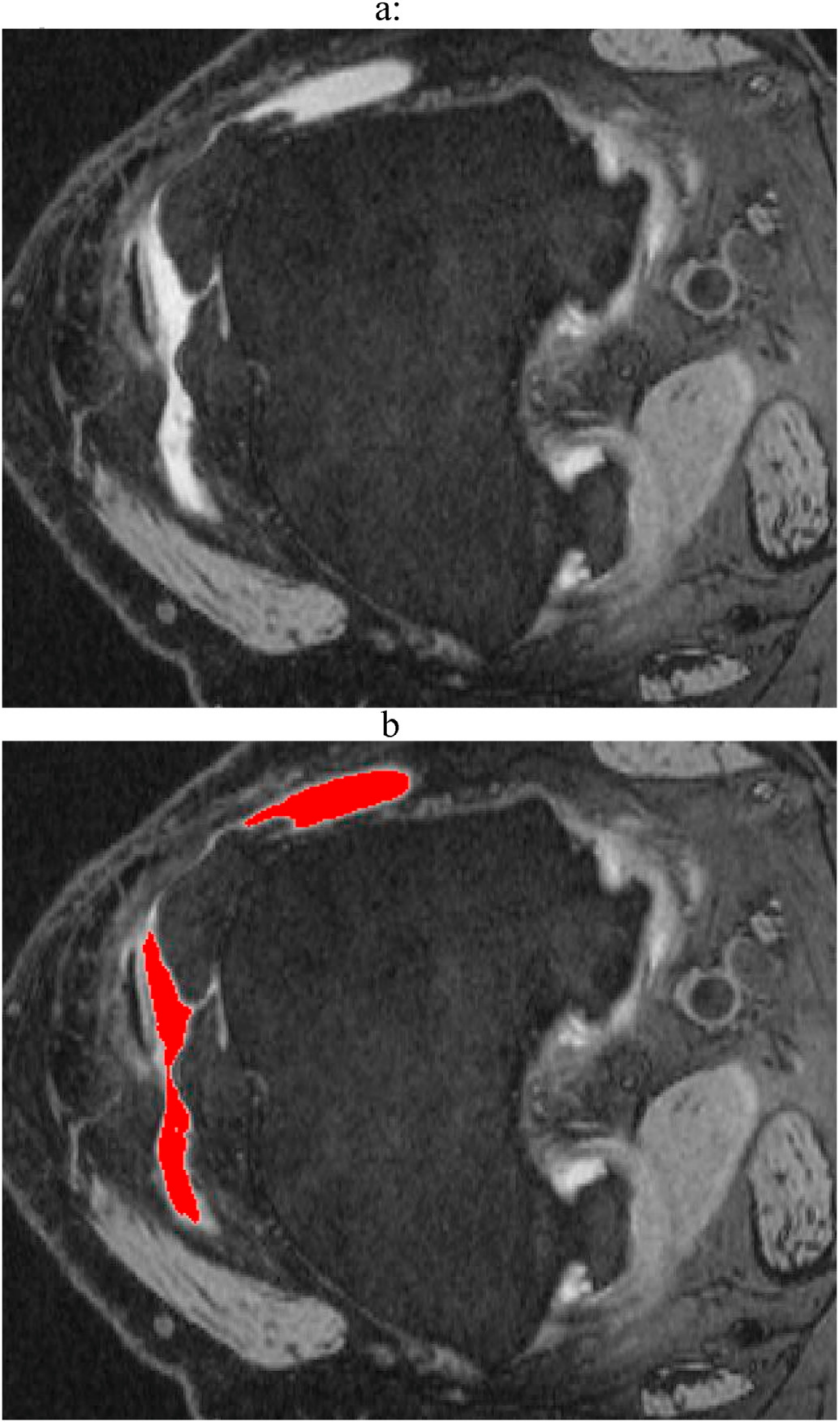
Example of the software joint effusion-synovitis (ES) measurement on MRI (a) Axial MR image of the knee at the level of the suprapatellar bursa depicting bright increased signal intensity fluid bursa fluid and edema. (b) The computer uses a thresholding method to identify the regions of joint effusion/synovitis which are shaded in red.

Non contrast axial 3 ​T DESS and sagittal TSE FS MR images of the knee (all performed on four identical Siemens Trio MR, Erlanger, Germany) were used for both ES and HS assessments respectively with separate semi-automated software tools developed for ES and HS. The ES software procedure was based on a variable grayscale threshold and region growing algorithms (Fig. 1). The threshold level was selected by the reader using a graphical user interface (GUI) such that highlighted regions were judged to best cover the true area of effusion; The total effusion volume (V_Eff_) in the patella region was measured by summing the effusion area across all assessed slices. Using the GUI, the reader was able to vary the threshold level and select regions that were judged to cover the joint fluid. Since the level of patella coverage was inconsistent in these data, we also calculated a normalized ES volume (V_Norm_) defined as the total volume divided by the number of slices in the patella region.

The Hoffa’s effusion software procedure consisted of manually drawing 4 regions of interest (ROIs) of the Hoffa’s fat pad on Slices i-3, i-1, i+1 and i+3, where Slice i was located at the center of the patella in the sagittal view. The measurement, X_Hoff_, was based on the volume of voxels in each ROI that exceeded a threshold determined by sampling a region of interest located approximately in the center of the femoral head. This component of the software was trained on an independent subset of the FNIH data, which consisted of varying several controlling parameters until maximum correlation with MOAKS was achieved.

Our study employed two readers: Reader 1 (SH) was a trained orthopedic surgeon and research fellow with knowledge of anatomy, pathology and radiological principles and 3 years of experience in his field; Reader 2 (SES) was a board-certified musculoskeletal radiologist with 19 years of experience. Reader 1 was responsible for reading the 301 scans to measure V_Eff_, while Reader 2 performed the X_Hoff_ measurements.

Fifty MR images from a subset of the 299 unused FNIH participants (independent data set) were used to train the readers and to optimize the X_Hoff_ algorithm. Demarcation of the regions of fluid/inflammation for ES was trialed by both readers in consensus on the independent data set prior to initiation of the study to ensure adequate education by the experienced MSK radiologist and to provide consistency. Similar training was also used to determine the regions of measurement for Hoffa’s fat pad by identifying the appropriate landmarks for each anatomical region.

Similarly, the suprapatellar bursa anatomic landmarks were identified from the superior suprapatellar bursa to the level of the inferior patella after which the reader selected the thresholding parameter to allow appropriate segmentation of the increased signal intensity. Reader judgement was used to confirm regions of increased signal intensity and potentially reject areas of irrelevant signal and make any necessary edits to the program drawn contours to ensure that the segmentation was correct.

Criterion validation was assessed through comparison with MOAKS. We used the central imaging assessment subsample of OAI participants available online (https://nda.nih.gov/oai/) who had had been scored by an experienced MSK radiologist. Both Readers 1 and 2 were blinded to the participant ID and MOAKS scoring.

Reliability was assessed using a sample of 30 participants read by both readers for both measurement types and the paired reads were separated by 4 weeks in order to avoid recall bias. The samples were randomly selected but weighted such that we obtained as uniform a distribution in MOAKS scores as possible. The measurement time for each method was also recorded.

### 2.3 Statistical analysis

The correlation to MOAKS scoring and reader precision were quantified using the Spearman correlation test and intra-class correlation coefficients (ICCs) respectively. Clinical validity was assessed using an analysis logistical regression analysis comparing cases to controls with the area under the curve (AUC) as the statistical metric. This test also allowed us to compare the quantitative method directly to MOAKS scoring using an independent metric.

## 3 Results

### 3.1 Participant characteristics

The 301 participants included in the analysis were 63% female and 96% Caucasian, with an average age of 62.0 and mean BMI of 26.7. Kellgren-Lawrence (KL) grades were distributed as follows: KL0: N ​= ​19, KL1: N ​= ​55, KL2: N ​= ​159, KL3: N ​= ​61, KL4: N ​= ​7. The MOAKS scores for ES were distributed as follows, 0: N ​= ​111, 1: N ​= ​131, 2: N ​= ​53, 3: N ​= ​6 and for HS were: 0: N ​= ​139, 1: N ​= ​135, 2: N ​= ​24, 3: N ​= ​3.

### 3.2 Concurrent validity: associations between quantitative methods and MOAKS scoring

**Fig. 2:**
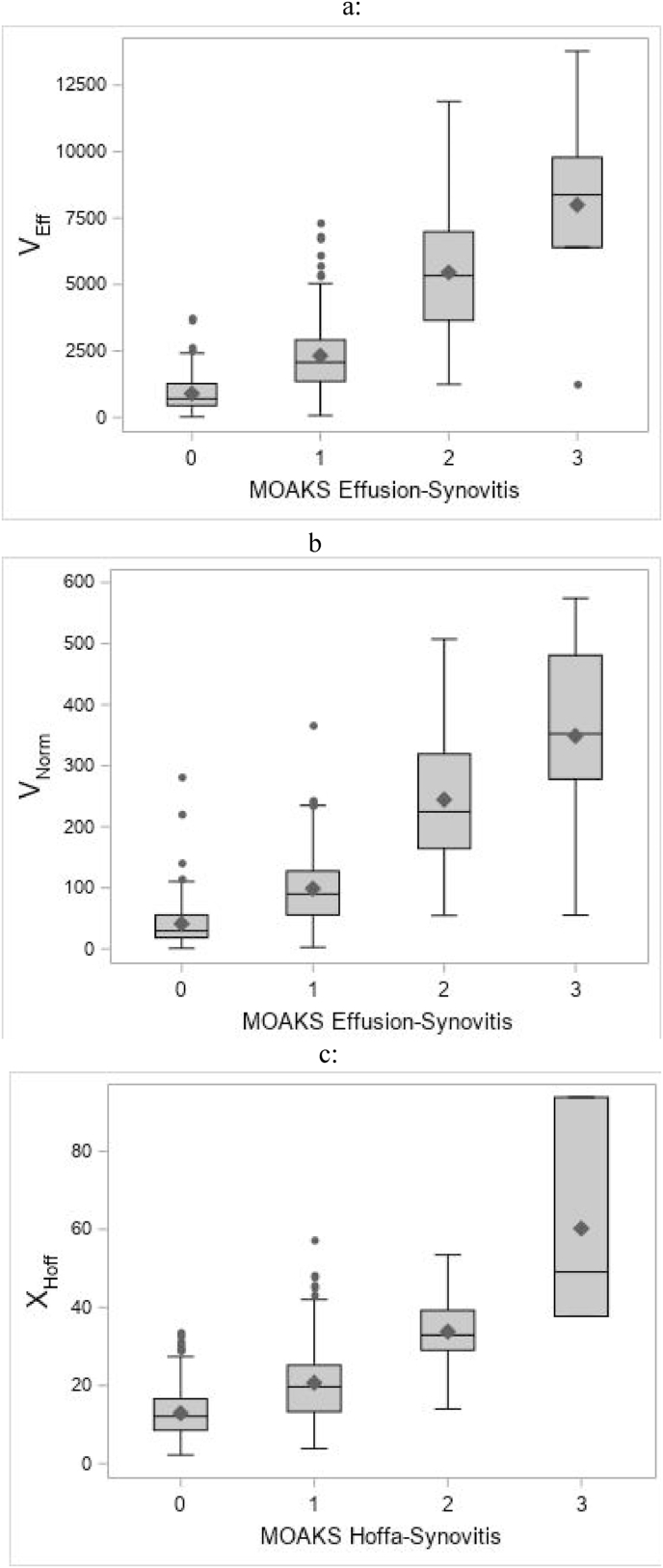
Whisker plots comparing (a) V_Eff_, (b) V_Norm_, and (c) X_Hoff_ to their corresponding MOAKS score.

Segmented ES volumes were highly correlated with the MOAKS scores (r ​= ​0.74). HS measurements were also correlated with the MOAKS scores (r ​= ​0.55) although less so than for ES. Fig. 2 shows whisker plots of the three software measures versus the corresponding MOAKS grade.

### 3.3 Clinical construct validity

Table 1a, Table 1ba and 1b gives the results for the FNIH case-control analysis. In general, we found similar AUC values for the volumetric measures compared to MOAKS scoring with a diminished association for the Hoffa’s measurement. V_Eff_ and V_Norm_ performed equally well.

**Table 1a:** AUC values with p values in parentheses and 95% confidence intervals (CI’s) in square brackets for MOAKS, software effusion/synovitis volume V_Eff_ and normalized software ES volume V_Norm_. The 95% CI’s were computed from bootstrap resampling with 500 replicates.

	Primary analysis Cases: Group 1. Controls: Groups 2, 3, and 4	Radiographic analysis Cases: Group 1 and 2. Controls: Groups 3 and 4	Pain analysis Cases: Group 1 and 3. Controls: Groups 2 and 4
MOAKS ES	0.54 (0.64) [0.50, 0.59]	0.57 (0.05) [0.51, 0.63]	0.52 (0.85) [0.50, 0.58]
V_Eff_	0.55 (0.14) [0.48, 0.62]	0.60 (<0.01) [0.53, 0.66]	0.52 (0.50) [0.48, 0.60]
V_Norm_	0.54 (0.08) [0.49, 0.62]	0.59 (<0.01) [0.53.0.66]	0.52 (0.67) [0.48, 0.59]

**Table 1b:** AUC values and p values in parentheses for MOAKS and software HS scores X_Hoff_.

	Primary analysis Cases: Group 1. Controls: Groups 2, 3, and 4	Radiographic analysis Cases: Group 1 and 2. Controls: Groups 3 and 4	Pain analysis Cases: Group 1 and 3. Controls: Groups 2 and 4
MOAKS HS	0.62 (<0.01) [0.54, 0.66]	0.64 (<0.01) [0.59, 0.70]	0.55 (0.35) [0.50, 0.60]
X_Hoff_	0.57 (0.18) [0.49, 0.63]	0.60 (0.02) [0.54, 0.66]	0.53 (0.32) [0.49, 0.59]

### 3.4 Reliability and reader time

For intra-reader reliability, scans for ES were assessed twice by Reader 1 while HS scans were read twice by Reader 2. For both V_Eff_ and V_Norm_, the ICC values for intra- and inter-reader reliability were 0.83 and 0.95, respectively. For X_Hoff_, the ICC values for intra- and inter-reader reliability were 0.98 and 0.96, respectively. The reader time was less than 15 ​min per knee for both methods combined.

## 4 Discussion

Our study provides evidence for the validity and reliability of a semi-automated quantitative method to measure ES volume and to characterize HS. V_Eff_ and X_Hoff_ are both strongly correlated with their corresponding MOAKS scores. The reader precision was excellent, and the case-control analysis demonstrated similar clinical validity of the metrics compared to the MOAKS scores.

To our knowledge, we are the first to perform both ES and HS evaluation with a quantitative software method. One study reported a semi-automated method to measure ES volume with an average reader time of 18 ​min [18] and a second quoted 10–15 ​min per scan [19]. An earlier study in 2010 by Li et al. demonstrated validation of a fully automated system based on MRI for the quantification of ES volume in OA patients in 25 patients, but the overall timing was not reported [21]. The largest and most recent study of 4115 OAI participants by Wang et al. (2019) utilized a fully automated system of evaluation of ES using DESS sequences at baseline and at 1 year which supported prior data from other semi-quantitative MR OA studies and concluding that an increase in effusion volume over one year was associated with increased medial femoral tibial cartilage volume loss, progression of radiographic OA and increased risk of total knee arthroplasty [21]. No calculated timeframe for the ES evaluation was provided in this study. Our method requires an average reader time of less than 15 ​min to measure of both ES and HS.

We found a substantially lower correlation to the MOAKS score for X_Hoff_ compared to V_Eff_ and Table 1b suggests that the HS measurement may have a lower association with the case control status compared to MOAKS. Both of these results could be a consequence of insufficient algorithm optimization. Alternatively, the results may be due to issues of intra- and inter-rater reliability of the MOAKS readings where the intra-rater kappa and inter-rater kappa for HS were 0.90 and 0.72 respectively, whereas for HS they were 0.42 and 0.70, respectively [17]. Going forward, a re-optimization of the HS software using a new larger training data set and a different optimization target may improve performance.

We also observed reduced reader precision for V_Eff_ versus X_Hoff_. A post-hoc assessment of the data revealed that a single outlier was responsible for a large reduction of the correlation. With this outlier removed, the ICC values were 0.97 and 0.99 for the inter- and intra-reader precision, respectively. For future studies, additional reader training may mitigate this issue. However, the other results of our study reinforce the validity of the V_Eff_ measurement and indicate that Reader 1 was properly trained and able to perform the assessments properly.

The reason to investigate V_Norm_ was based on the hypothesis that the normalized volume could correct for the inconsistent patella coverage in the axial scans and to adjust for knee size. Interestingly, there were negligible differences in performance between V_Eff_ and V_Norm_. This result is perhaps less surprising for precision and comparison to MOAKS since all readers (software as well as the MOAKS reader) used the same data for evaluation. However, this also held true for the clinical validity component of our study. These data suggest that it may be possible to assess effusion volume with fewer images, perhaps skipping slices as we did for X_Hoff_. This potential time-saving approach requires further study.

The methods proved efficient with an average reading time of 15 ​min per knee. The measurements were performed by a fellowship trained MSK radiologist (SES) with training in MOAKS and automated BML and ES scoring, and by another trained reader (SH) with anatomic knowledge in orthopedics and training from expert radiologists confirming that this method could be performed by a less-skilled reader trained by an expert radiologist.

Strengths of this study include the relatively large patient cohort, blinded readings, and standardized methods used for data acquisition. We also performed a comprehensive evaluation including criterion and clinical validity, as well as precision and reader time. Both methods were evaluated in a sample population with a range of OA severity. Future work will include validation against additional clinical and structural outcomes such as total knee replacement and cartilage loss in future observational studies or clinical trials.

This study had several limitations. All images were non-contrast intermediate weighted 3D DESS or TSE images performed on a specific Siemens 3 ​T magnet type. No other sequences were evaluated. We did not use the full 600 subject FNIH data set; however a sample size of 301 is relatively large and was chosen to exactly match the group distribution of the full cohort. The remaining 299 were unavailable for evaluation by the software since many were used for development and training. While fast, a reader time of 15 ​min implies a considerable and feasible effort for studies involving thousands of images. Future work will be directed at substantially lowering the reader time using deep learning (DL) methods. The current level of efficiency will allow us to generate substantial training data that can be used to develop DL algorithms.

In conclusion, we have validated an efficient and rapid software tool to quantify ES and HS in KOA subjects using MRI that is highly accurate for ES but less so for HS. This method can provide a quick and proven quantitative measure of these important structural variables, allowing faster assessment in future larger scale studies of the knees in OA evaluation.

## Author contributions:

Stacy E. Smith: Conceptualization and design, Methodology, Formal analysis, Investigation, Data curation, Writing – original draft, Writing-review and editing, Visualization, Final approval of the version to be submitted. Shayan Hosseinzadeh: Conceptualization and design, Methodology, Formal analysis, Investigation, Technical support Writing – review and editing, Final approval of the version to be submitted. Troy Maetani: Conceptualization, Investigation, Acquisition of data, Methodology, Formal analysis and interpretation of data, Technical support, Writing-review and editing, Final approval of the version to be submitted. Shilpa Pandey: Conceptualization, Methodology, Validation, Investigation, Technical support, Writing – review & editing, Final approval of the version to be submitted. C. Kent Kwoh: Conceptualization, Methodology, Investigation, Writing – review & editing, Final approval of the version to be submitted.Jeffrey Duryea: Conceptualization, Methodology, Formal analysis and interpretation of data, Statistics, Investigation, Resources, Writing – review and writing, Project administration and Supervision, Funding acquisition, Final approval of the version to be submitted.

## Sponsor

NIH AR071409: Tracking Treatable Tissues: Change in qMRI Biomarkers and Future Cartilage Loss.

## Authorship

All authors should have made substantial contributions to all of the following: (1) the conception and design of the study, or acquisition of data, or analysis and interpretation of data, (2) drafting the article or revising it critically for important intellectual content, (3) final approval of the version to be submitted. By signing below each author also verifies that he (she) confirms that neither this manuscript, nor one with substantially similar content, has been submitted, accepted or published elsewhere (except as an abstract). Each manuscript must be accompanied by a declaration of contributions relating to sections (1), (2) and (3) above. This declaration should also name one or more authors who take responsibility for the integrity of the work as a whole, from inception to finished article. These declarations will be included in the published manuscript. Acknowledgement of other contributors.

All contributors who do not meet the criteria for authorship as defined above should be listed in an acknowledgements section. Examples of those who might be acknowledged include a person who provided purely technical help, writing assistance, or a department chair who provided only general support. Such contributors must give their consent to being named. Authors should disclose whether they had any writing assistance and identify the entity that paid for this assistance.

## Role of the funding source

Authors should declare the role of study sponsors, if any, in the study design, in the collection, analysis and interpretation of data; in the writing of the manuscript; and in the decision to submit the manuscript for publication. If the study sponsors had no such involvement, the authors should state this.

## Studies involving humans or animals

Clinical trials or other experimentation on humans must be in accordance with the ethical standards of the responsible committee on human experimentation (institutional and national) *and* with the Helsinki Declaration of 1975, as revised in 2000. Randomized controlled trials should follow the Consolidated Standards of Reporting Trials (CONSORT) guidelines and be registered in a public trials registry.

Studies involving experiments with animals were in accordance with institution guidelines

## Role of funding sources

The OAI is a public-private partnership comprised of five contracts (N01-AR-2-2258; N01-AR-2-2259; N01-AR-2-2260; N01-AR-2-2261; N01-AR-2-2262) funded by the 10.13039/100000002National Institutes of Health, a branch of the 10.13039/100000016Department of Health and Human Services, and conducted by the OAI Study Investigators. Private funding partners include 10.13039/100004334Merck Research Laboratories; 10.13039/100008272Novartis Pharmaceuticals Corporation, GlaxoSmithKline; and 10.13039/100004319Pfizer, Inc. Private sector funding for the OAI is managed by the Foundation for the 10.13039/100000002National Institutes of Health.

The private funding partners had no role in the study design or in the collection, analysis, or interpretation of the data, the writing of the manuscript, or the decision to submit the manuscript for publication. Publication of this article was not contingent upon approval by the private funding partners.

This manuscript was prepared using an OAI public use data set and does not necessarily reflect the opinions or views of the OAI investigators, the 10.13039/100000002NIH, or the private funding partners.

## Declaration of competing interest

The authors declare that they have no conflict of interest.
